# Oral Viral DNA Profiling in Obesity, Adenomatous Polyposis, and Colorectal Cancer Identifies Human β-Papillomavirus Types as Potentially Sex-Related and Modifiable Cancer Risk Indicators

**DOI:** 10.3390/cancers17183024

**Published:** 2025-09-16

**Authors:** Veronica Fertitta, David Israel Escobar Marcillo, Grete Francesca Privitera, Manuela Del Cornò, Valeria Guglielmi, Annamaria Agnes, Barbara Varano, Luca Colangeli, Lorenzo Ferri, Sandrine McKay-Chopin, Paolo Sbraccia, Roberto Persiani, Alfredo Pulvirenti, Zdenko Herceg, Massimo Tommasino, Tarik Gheit, Paola Fortini, Lucia Conti

**Affiliations:** 1 Department of Environment and Health, Istituto Superiore di Sanità, 00161 Rome, Italy; veronica.fertitta@unicatt.it (V.F.); escobard@iarc.who.int (D.I.E.M.); paola.fortini@iss.it (P.F.); 2 Epigenomics and Mechanisms Branch, International Agency for Research on Cancer (IARC), 69007 Lyon, France; chopin@iarc.fr (S.M.-C.); hercegz@iarc.who.int (Z.H.);; 3 Bioinformatics Unit, Department of Clinical and Experimental Medicine, University of Catania, 95123 Catania, Italy; grete.privitera@unict.it (G.F.P.); alfredo.pulvirenti@unict.it (A.P.); 4 Center for Gender-specific Medicine, Istituto Superiore di Sanità, 00161 Rome, Italy; manuela.delcorno@iss.it (M.D.C.); barbara.varano@iss.it (B.V.); 5 Internal Medicine Unit and Obesity Center, University Hospital Policlinico Tor Vergata, 00133 Rome, Italy; valeria.guglielmi@uniroma2.it (V.G.); luca.colangeli@ptvonline.it (L.C.); sbraccia@med.uniroma2.it (P.S.); 6 Department of Medical and Surgical Sciences, Fondazione Policlinico Universitario Agostino Gemelli IRCCS, 00168 Rome, Italy; annamaria.agnes@policlinicogemelli.it (A.A.); lorenzo.ferri@guest.policlinicogemelli.it (L.F.); roberto.persiani@unicatt.it (R.P.)

**Keywords:** viruses, infections, colorectal cancer, obesity, colon adenomatous polyps, cancer risk factors, cancer biomarkers, oral cavity, HPV genotyping

## Abstract

**Background/Objectives**: Colorectal cancer (CRC) is the third most common cancer and a leading cause of death worldwide. Identifying non-invasive, early indicators of CRC risk remains essential and could help reduce its health burden. Excess adiposity and chronic inflammation are major predisposing factors for precancerous adenomatous polyposis (AP) and CRC, while diet- or surgery-induced weight loss was associated with a reduced risk. Viral infections also represent cancer risk factors through direct or synergic mechanisms, though no definitive causal link has been established for CRC. Moreover, interest is growing on the role of oral viruses as predictors of disease. **Methods**: In this study, highly sensitive and specific Luminex-based screening assays were used to perform a comprehensive characterization of oral infections by Human Herpes (HHV), Polyoma (HPyV) and Papilloma (HPV) Viruses in CRC patients (N = 50), healthy controls (N = 46; normal weight, NW = 26; overweight, OW = 20), and high-risk individuals with obesity (N = 35) or adenomatous polyposis (AP, N = 22). **Results**: We observed increased HPyV prevalence in AP, and higher single and multiple β-HPV infection rates in AP and CRC compared to controls. A panel of β-HPV genotypes, including oncogenic HPV5, was overrepresented in CRC and high-risk groups, and some of them showed an association with the male sex. The prevalence of most infections decreased in the obese cohort following bariatric surgery, alongside weight loss and reduction of inflammatory markers. Furthermore, oral infections by viral types previously detected in CRC tissue and adjacent mucosa also declined after surgery. **Conclusions**: Altogether, these findings suggested a role for oral β-HPV types as potential sex- and lifestyle-related, modifiable indicators of cancer risk.

## Simple Summary

Colorectal cancer (CRC) is the third most common cancer and a leading cause of death worldwide. Most CRC cases are diagnosed at an advanced stage, highlighting the need for effective early detection methods. In this regard, easily accessible biomarkers can help clinicians achieve this goal. Multiple factors, such as diet, environment, age, and genetics, are associated with CRC development. Emerging evidence indicates a potential role of viral infections, although these associations are incompletely understood. We found that human herpes, polyoma, and papilloma (HPV) viruses are common in the oral cavity of healthy individuals, and their prevalence increases in CRC patients, as well as in high-risk populations, such as individuals with obesity or colon adenomatous polyposis. Furthermore, surgery-induced weight loss in subjects with obesity is associated with a reduction in some beta-HPV types, arguing for their role as potential lifestyle-related early indicators of cancer risk.

## 1. Introduction

Colorectal cancer (CRC) is a major public health threat, being the third most common cancer and a leading cause of death worldwide, with incidence rates under the age of 50 expected to increase in the coming years [1]. Current screening methods are limited by low sensitivity or invasiveness, and studies aimed at investigating blood-based biomarkers remain insufficient [2]. The identification of non-invasive indicators able to predict CRC risk or early-stage disease and to guide prevention would help reduce its negative health impact.

The increasing incidence of CRC reflects the overweight and obesity epidemics and the adoption of Western lifestyles [3,4]. Epidemiological and preclinical studies have shown that excess adiposity is a major predisposing factor for precancerous adenomatous polyposis (AP) and CRC, with important implications for prevention [4,5]. Indeed, CRC is highly preventable through lifestyle modifications, and both diet- and surgery-induced weight loss have been associated with reduced risk [6,7]. The obesity–CRC axis is complex and multifaceted. Adipose tissue and systemic chronic inflammation, oxidative imbalance, and immunosuppression characterize both conditions, and several studies have reported the impairment or depletion of immune regulators and cell subsets involved in the early surveillance of cancer as well as in the control of microbial infections [8,9,10,11]. In this regard, increased susceptibility to and reduced clearance of respiratory virus infections, as well as an association with nosocomial microbial infections, have been repeatedly reported in overweight (OW) and obese individuals [12,13].

Viral infections also represent risk factors for several cancers, contributing to approximately 15% of all cancer cases worldwide [1,14]. In addition to their direct oncogenic properties, viruses can act in synergy with other risk factors to initiate or sustain carcinogenesis [14]. Several studies have attempted to unravel the potential link between virus infection and cancers of the gastrointestinal tract, and viral DNA and proteins have been detected in tumor tissues [15]. Human herpes (HHV), polyoma (HPyV), and papilloma (HPV) virus DNA has been detected in CRC tissues as well as in precancerous colon lesions, although a causal link with intestinal carcinogenesis has not been established yet [16]. Epstein–Barr virus, John Cunningham polyomavirus, and the high-risk mucosal HPV types (mainly HPV16 and HPV18), as well as co-infections, have been consistently reported to be prevalent in CRC tissues and associated with cancer risk [15,16]. In addition, co-infection with multiple high-risk α-HPV subtypes has been correlated with advanced tumor stages [17]. More recently, combined PCR protocols, NGS, and Luminex bead-based assays were used to determine the presence of a broad spectrum of mucosal (α) and cutaneous (β and γ) HPV types in CRC tissue samples, leading to the identification of viral DNA from different β- and γ-HPV genotypes both in the tumor tissue and in the adjacent normal mucosa [18].

Among HPV types, α and β have garnered significant attention due to their association with human health. While the role of high-risk mucosal α-HPV types is well established in anogenital and oropharyngeal cancers, cutaneous β-HPV genotypes are suspected to contribute to the development of non-melanoma skin cancers, in synergy with other risk factors. This group of viruses was shown to act as cofactors or facilitators of cancer development at the early stages of skin carcinogenesis, suggesting a hit-and-run mechanism [19,20,21,22]. Moreover, a role in promoting co-infection or favoring the persistence of high-risk α-HPV has been hypothesized for cutaneous β-HPV [23]. Similarly, DNA viruses like HHV and HPyV, commonly found in the human population, can contribute to cancer development [24]. Due to the crucial role of the immune system in infection clearance, viral persistence or reactivation and increased long-term cancer risk have been reported in individuals with congenital or acquired immunodeficiencies [25,26,27].

Over the past decade, scientific interest has increased regarding the prevalence and distribution of β-HPV in non-cutaneous anatomical sites [28], such as the genital, anal, and oral mucosa [29,30,31,32].

Oral β-HPV prevalence in the healthy population can reach up to 60%, whereas higher viral prevalence and multiple infections have been reported in cohorts of immunocompromised or highly exposed individuals, correlating with impaired immune functions [28,33,34,35,36,37,38,39]. Moreover, the oral prevalence of specific β-HPV genotypes was consistently found to increase in individuals with oral and oropharyngeal cancers (HPV5, HPV24, HPV122) or with precancerous lesions (HPV120, HPV124) compared to healthy controls [40]. Results from a recent study indicated that, unlike α- and γ-HPV, the oral distribution of β-HPV is not substantially affected by sociodemographic and behavioral characteristics such as age, sexual behavior, oral health status, smoking, and alcohol consumption [34].

Due to their wide distribution across different anatomical sites and their acquisition early in life, cutaneous HPV types can be considered part of the commensal flora or microbiota [41]. Intestinal dysbiosis is a common feature in individuals with OW or obesity, as well as in patients with CRC or precancerous lesions. Over the past decade, research has highlighted profound alterations in the composition of gut bacteria in both CRC and high-risk conditions. More recently, an oral–gut microbiota axis has been uncovered, as oral microorganisms have been shown to migrate to the gut, where they may promote carcinogenesis [42]. The study of the oral microbiome is an emerging area of investigation that is revealing the potential predictive role of oral microorganisms in various pathologies, including CRC [43,44]. While most studies have focused on bacteria, the virome is now recognized as an essential component of microbiota. Apart from scattered observations of CRC-associated changes in the gut virome, mainly bacteriophages [45,46], studies investigating the risk-predictive potential of human viruses in the oral cavity, as well as their role as cofactors in CRC pathogenesis, are lacking. Moreover, few longitudinal studies have examined how immune, hormonal, or lifestyle factors influence oral virus persistence. Although changes in lipid metabolism have been shown to strongly influence viral infections [47], the role of body weight or adiposity in viral persistence has not been considered in previous studies. Chronic α-HPV infection has recently been associated with oral inflammation [48]; however, whether the chronic low-grade systemic inflammation characterizing OW/obesity and obesity-associated diseases can impact oral viral infections has yet to be explored.

In this pilot study, we employed highly sensitive and specific Luminex-based screening assays to investigate the prevalence of HHV, HPyV, and HPV infections in the oral cavity of CRC patients and high-risk subject groups, including individuals with OW, obesity, or AP. We report for the first time an increased prevalence of HPyV infections in subjects with AP, as well as increased β-HPV prevalence and multiple infections in CRC patients and high-risk individuals compared to healthy controls. Furthermore, specific β-HPV genotypes were overrepresented among CRC patients, individuals with obesity or AP, as well as in OW compared to normal-weight (NW) controls. Notably, surgery-induced weight loss in the obese cohort was associated with a reduction in the prevalence of these viral genotypes, suggesting their potential role as lifestyle-associated, and thus modifiable, early indicators of cancer risk.

## 2. Materials and Methods

### 2.1. Study Populations

Four different subject cohorts were recruited in this study. A total of 50 individuals with colorectal cancer (CRC, male/female 27/23; mean age 70.6 ± 8.98 years; mean BMI 25.55 ± 3.6 kg/m^2^; tumor stage 0–I to IV according to the TNM classification) and 22 subjects with nonfamilial adenomatous polyposis (AP, male/female 17/5; mean age 65.4 ± 11.6 years; mean BMI 26.3 ± 3.46 kg/m^2^; polyps classified by number, size, and histology, low- or high-grade dysplasia) were recruited at the IRCCS Fondazione Policlinico Universitario Agostino Gemelli (Rome, Italy). Subjects were enrolled at diagnosis, before receiving any therapeutic treatment. Exclusion criteria were a family history of CRC or AP, diabetes, pregnancy, severe mental disorders, active infections, chronic inflammatory diseases, immunomodulating treatment, hormone therapy, or use of antibiotics, probiotics, and anti-inflammatory drugs in the last 30 days. The male sex overrepresentation in the AP group was due to the known higher incidence of AP in men, combined with the low participation of women in the CRC screening program of the reference clinical center. All participants underwent an anamnestic interview, and anthropometric and clinical parameters were recorded.

A total of 35 subjects affected by severe obesity (32 females; mean age 52.6 ± 10.84 years; mean BMI 44.75 ± 6.49 kg/m^2^) eligible for bariatric surgery (BS) were enrolled at the Obesity Center of the University Hospital “Policlinico Tor Vergata” (Rome, Italy). A total of 28 out of 35 enrolled subjects completed the study and were included in the time-course analysis. Eligible patients met the criteria for BS and had a stable body weight in the 3 months preceding the study. The exclusion criteria were as follows: known medical history of diabetes, active infections, cancer, and other acute or chronic diseases; use of nonsteroidal anti-inflammatory drugs, glucocorticoids, antibiotics, prebiotics, and probiotics in the last month before BS. The female prevalence, according to most BS studies where men are underrepresented, was largely due to the significantly higher utilization of BS among eligible women. At enrolment, as well as at 6 (T6) and 12 (T12) months after BS, all patients underwent an anamnestic interview, and both anthropometric and clinical parameters were recorded. Body composition was determined by bioimpedance analysis. After BS, patients followed a specific diet, and the mean BMI dropped to 29.8 ± 5.1 kg/m^2^ at the end of the follow-up [49,50].

A total of 46 healthy individuals (male/female 25/21; mean age 51.7 ± 12.44 years; mean BMI 24.68 ± 2.7 kg/m^2^) were enrolled at the University Hospital “Policlinico Tor Vergata” (Rome, Italy) as controls. The exclusion criteria were the same as those applied to the cohort of subjects affected by obesity. Demographic and anthropometric parameters were collected.

The study was conducted in accordance with the guidelines of the Declaration of Helsinki and was approved by the Ethical Committees of Istituto Superiore di Sanità (ISS-15/03/16-n.173/16 and AOO-ISS-24/05/19-0016145). All participants in the study signed informed consent forms and filled out a questionnaire that collected information about their family and medical history, current medication use, smoking, dietary, and physical activity habits. Sample collection and processing were performed by assigning a specific code to each subject to maintain anonymity, in compliance with the EU General Data Protection Regulation.

### 2.2. Sample Collection

Oral rinse and gargle samples were collected at enrolment from all subject cohorts. For subjects with obesity, samples were also collected at 6 and 12 months after BS. Participants rinsed and gargled with 15 mL of sterile saline solution (0.9% NaCl) for 30 s. Samples were then transferred into sterile tubes containing PreservCyt Solution (Hologic. Inc., Malborough, MA, USA), and centrifuged at 3000× *g* for 10 min at 4 °C to remove the mouthwash. The cell pellet was suspended in 1 mL of the same solution and stored at −80 °C for subsequent viral DNA testing. All participants were asked not to eat or brush their teeth for at least 1 h before sampling. Whole blood samples were also collected from subjects with obesity at baseline and at 6 and 12 months after BS for plasma separation.

### 2.3. DNA Extraction from Oral Rinse and Gargle Samples

Samples were homogenized twice for 30 s at 6000 rpm using MagNA Lyser Green Beads (Roche, Monza, Italy), and DNA was isolated using the Qiagen BioRobot EZ1 Advanced XL with the EZ1 Advanced DSP Virus Card, according to the manufacturer’s instructions (Qiagen, Venlo, The Netherlands). The concentration of DNA was determined using the Quant-iT PicoGreen dsDNA Assay Kit (Invitrogen, Waltham, MA, USA).

### 2.4. Viral DNA Detection Assay

Type-specific multiplex genotyping (TS-MPG) assays, based on multiplex polymerase chain reaction (PCR) and bead-based Luminex technology (Luminex Corp., Austin, TX, USA), were used to analyze the presence of DNA from human papillomavirus (HPV), polyomavirus (HPyV), and herpesvirus (HHV) genotypes. Using 10 µL of each DNA extract, multiplex PCRs were performed using specific primers targeting 21 α-HPV (HPV6, 11, 16, 18, 26, 31, 33, 35, 39, 45, 51, 52, 53, 56, 58, 59, 66, 68, 70, 73, and 82), 45 β-HPV types (HPV5, 8, 9, 12, 14, 15, 17, 19, 20, 21, 22, 23, 24, 25, 36, 37, 38, 47, 49, 75, 76, 80, 92, 93, 96, 98, 99, 100, 104, 105, 107, 110, 111, 113, 115, 118, 120, 122, 124, 143, 145, 150, 151, 152, and 159), 52 γ-HPV types (HPV4, 48, 50, 60, 65, 88, 95, 101, 103, 108, 109, 112, 116, 119, 121, 123, 126, 127, 128, 129, 130, 131, 132, 133, 134, 148, 149, 156, 161, 162, 163, 164, 165, 166, 167, 168, 169, 170, 171, 172, 173, 175, 178, 179, 180, 184, 197, 199, 200, 201, 202, and SD2 (PMID: 23554892)), 13 HPyV types (BKPyV, WUPyV, KIPyV, MCPyV, JCPyV, HPyV6, HpyV7, HPyV9, HPyV10, TSPyV, HPyV12, LIPyV, and SV40), and 10 HHV [HSV1, HSV2, HHV3 (HZV) EBV1, EBV2, CMV, HHV6A, HHV6B, HHV7, and HHV8 (KSHV)]. In addition, two primers for human beta-globin were used to assess the quality of the DNA extract. After PCR amplification, 10 μL of each reaction was analyzed by multiplex genotyping using a Luminex-based assay, as previously described [51,52,53]. The results are reported as median fluorescence intensity (MFI). For each probe, MFI values obtained without PCR product in the hybridization mixture were considered background. The cutoff was defined as 1.1 times the median background value plus 5 MFI, as previously described [51]. The MFI data used for subsequent statistical analyses are provided in Supplementary Data S1–S5.

### 2.5. Quantification of Plasma Mediators

Plasma TNF-α, IL6, and leptin levels were evaluated using a Human High-Sensitivity Magnetic Luminex Performance Assay kit (R&D Systems, Minneapolis, MN, USA). Bio-Plex Manager software (version 6.1, Bio-Rad Laboratories, Inc., Segrate, MI, Italy) was used for data analysis. Plasma levels of adiponectin were determined by ELISA (R&D Systems, Minneapolis, MN, USA), according to the manufacturer’s instructions. Droplet digital PCR (Bio-Rad QX 200, Bio-Rad Laboratories, Inc., Segrate, MI, Italy) was performed to evaluate the levels of circulating cell-free mitochondrial DNA (ccf-mtDNA) fragments in plasma samples. The analysis was carried out using 0.5 µL of plasma. MT-ND2 and RPLP0 TaqMan probes were used to detect mitochondrial and nuclear DNA, respectively (cat. no. 4331182; Life Technologies, Carlsbad, CA, USA).

### 2.6. Statistical Analysis

The HPV, HPyV, and HHV prevalence is expressed as the percentage of all subjects tested and represents the prevalence in both single and multiple infections. Samples with multiple infections are those with two or more viral types detected and were counted as positive for each type. The prevalence (n/N%) of any given virus genotype infection was calculated as the percentage of individuals who tested positive (n) for the genotype of interest out of the total number of individuals tested in each group (N).

All statistical tests were conducted employing R (v.4.2.0). The differences in prevalence between groups were tested with Fisher’s exact test, considering as significant only differences with a *p*-value < 0.05. The odds ratio (OR) was calculated with Fisher’s exact test to measure the association between the categorical variables. The 95% confidence interval (CI) was calculated to estimate the precision of the OR. The differences between age and BMI were assessed by a *t*-test since the population was normally distributed. Normality was assessed by the Shapiro–Wilk test. The possible confounding variables, such as smoking, age, and sex, were analyzed using a generalized linear model (glm) through the package MatchIt (v.4.7.2). Statistical tests for inflammatory mediators were performed using the Kruskal–Wallis or ANOVA tests (employing, respectively, the Wilcoxon signed-rank test and *t*-test for paired couples). The appropriate analysis was chosen through the Shapiro–Wilk test, analyzing the population distribution. All plots were generated using the R packages ggplot (v.3.5.1) and ggpubr (v.0.6.0).

## 3. Results

### 3.1. Comparative Analysis of Oral DNA Virus Profiles in Colorectal Cancer Patients and At-Risk Populations with Obesity or Nonfamilial Adenomatous Polyposis

The presence and distribution of selected oral human DNA viruses were investigated in patients with CRC (cancer group) and individuals with obesity or nonfamilial AP, both considered at-risk groups, in comparison to a healthy control group. Oral rinse and gargle samples were analyzed using a bead-based Luminex assay to detect viral DNA from HPV, HPyV, and HHV. In total, 153 samples were collected from 35 individuals with obesity, 22 with AP, 50 CRC patients, and 46 healthy controls. The anthropometric and clinical characteristics of the study groups are reported in Table 1.

**Figure 1:**
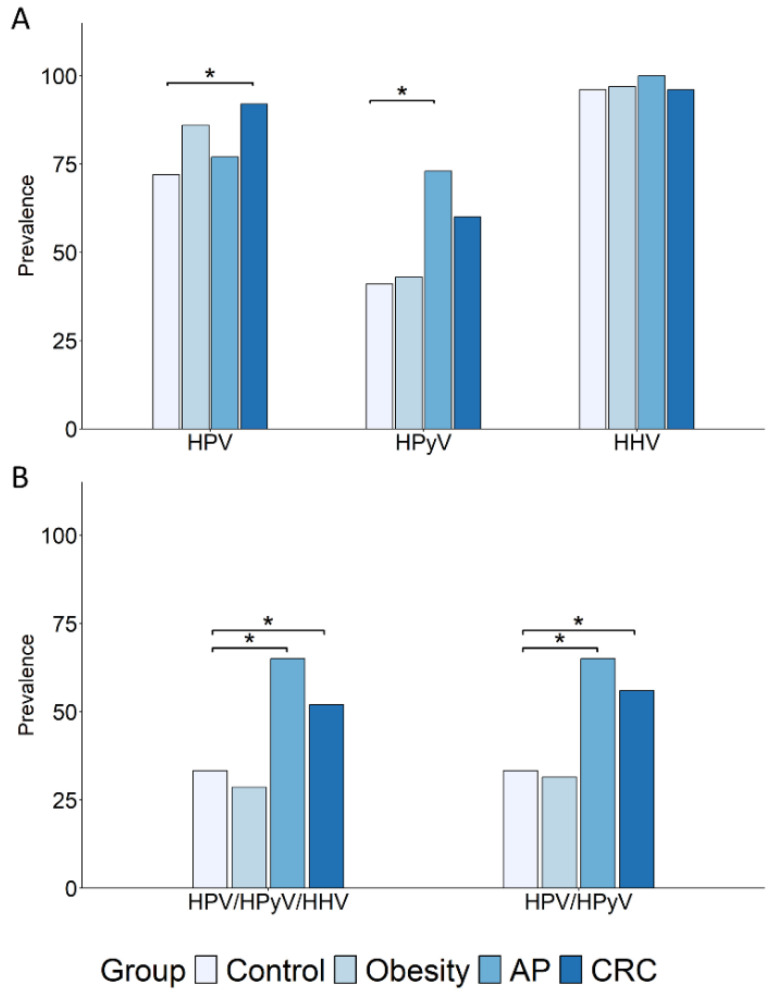
Prevalence of oral HPV, HPyV, and HHV infections and co-infections in subjects with obesity or AP and in CRC patients. The prevalence of HPV, HPyV, and HHV infections (**A**) and co-infections (**B**) in subjects with obesity (N = 35) or AP (N = 22), and in CRC patients (N = 50), is shown compared to the healthy control group (N = 46). Subjects harboring at least one infection for each viral family were included. Comparisons were performed using Fisher’s exact test, and statistical significance is indicated (* *p* < 0.05).

The analysis of virus families revealed a significantly higher prevalence of HPV infections, regardless of the genus, among CRC patients, and a trend toward increased prevalence in subjects with obesity, compared to controls (Figure 1A and Table 2). Conversely, the AP group exhibited a significantly higher frequency of HPyV infections compared to the control group (Figure 1A and Table 2). The prevalence of HHV infections exceeded 95% across all groups, with no significant differences observed (Figure 1A and Table 2). Notably, when co-infections involving any genotype within each viral family were analyzed, a markedly increased prevalence of HPV/HPyV/HHV and HPV/HPyV co-infections was observed in both the AP and CRC groups compared to the control and obesity groups (Figure 1B).

The analysis of HPyV family members allowed us to identify only two genotypes, HPyV6 and Merkel cell polyomavirus (MCPyV). As shown in Table 2, HPyV6 prevalence was significantly higher in the AP versus the control group, whereas MCPyV was more commonly detected among the CRC and AP groups.

For HHV, the most frequently detected genotypes across all groups were EBV1, whose prevalence was significantly higher in CRC patients compared to controls, HHV-6B, and HHV7 (Table 2).

### 3.2. The β-HPV Genus Is the Most Frequently Detected and Differentially Distributed Among Subject Groups

**Figure 2:**
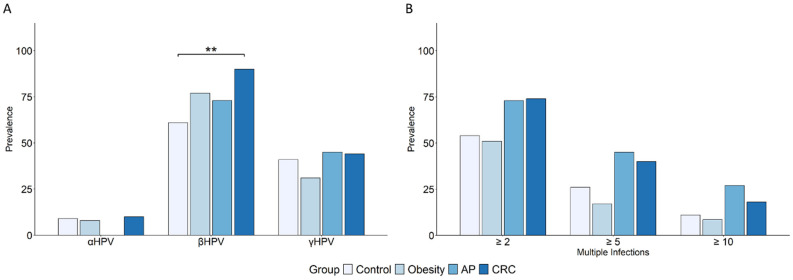
Prevalence of oral α-, β-, and γ-HPV infections and multiple β-HPV infections. The prevalence of α-, β-, and γ-HPV infections (**A**) and of multiple β-HPV infections (**B**) in subjects with obesity (N = 35), AP (N = 22), and CRC (N = 50) is reported in comparison to the control group (N = 46). Subjects harboring at least one infection for each viral genus (**A**) or at least two β-HPV infections (**B**) were included. Comparisons were performed using Fisher’s exact test, and statistical significance is indicated (** *p* < 0.01).

Within HPV, the β genus was the most frequently detected in the oral cavity across all subject groups (Figure 2A and Table 2). Notably, a significantly higher prevalence of β-HPV was observed among CRC patients compared to healthy controls. The high-risk obesity and AP groups also exhibited higher β-HPV frequencies, suggesting a possible association with cancer risk. In contrast, cutaneous γ-HPV types were less prevalent and showed no significant differences among groups (Figure 2A and Table 2). Regarding α-HPV, previously associated with CRC, no substantial differences were observed between patients and controls (Figure 2A and Table 2). High-risk α-HPV genotypes were rare and similarly distributed between the CRC and control groups. In individuals with obesity, higher frequencies of HPV16 (6.25% vs. 2%) and HPV31 (3.125% vs. 0%) were observed compared to controls, whereas no α-HPV was detected in the AP group.

Concerning β-HPV infections, the percentages of individuals with single-genotype infections in the control, obesity, AP, and CRC groups were 10.7%, 34.6%, 0%, and 15.6%, respectively. However, most subjects harbored at least two β-HPV genotypes (54%, 51%, 73%, and 74%, respectively). Based on the number of genotypes detected, subjects were categorized into subgroups with ≥2, ≥5, and ≥10 β-HPV infections. As for the above-reported HPV/HPyV/HHV co-infections, this analysis also revealed a higher proportion of multiple β-HPV infections in patients with colorectal neoplasia (≥2: 74 vs. 54%, *p* = 0.056, OR = 2.37, 95% CI: 0.93, 6.20; ≥5: 40 vs. 26%, *p* = 0.19, OR = 1.88, 95% CI: 0.73, 4.97; ≥10: 18 vs. 11%, *p* = 0.39, OR = 1.79, 95% CI: 0.49, 7.41) and in those with precancerous lesions (≥2: 73 vs. 54%, *p* = 0.19, OR = 2.21, 95% CI: 0.67, 8.21; ≥5: 45 vs. 26%, *p* = 0.16; OR = 2.33, 95% CI: 0.7, 7.78; ≥10: 27 vs. 11%, *p* = 0.15, OR = 3.02, 95% CI: 0.66, 14.47) compared to healthy controls (Figure 2B).

### 3.3. Impact of Tumor Stage and Precancerous Lesion Features on Oral Virus Prevalence

CRC patients at different tumor stages were included in the study (Table 1). To explore whether viral prevalence could be associated with tumor stages at diagnosis, we compared patient subgroups diagnosed at early, intermediate, and advanced/metastatic tumor stages. Supplementary Figure S1A,B show that no statistically significant differences were observed among stage 0–I, II, and III–IV patient subgroups in the prevalence of infections by HPV, HPyV, and HHV (A), as well as of β-HPV infection and multiple infections (B). The association of virus infections with the features of precancerous lesions in the AP group was also investigated. Subjects were subgrouped into high- and low-risk, based on the presence or absence of polyps with high-grade dysplasia, size greater than 10 mm, or villous histology (Table 1). As shown in Supplementary Figure S1C,D, no significant differences were observed between groups in the prevalence of infections by HHV, HPV, and β-HPV, whereas low-risk individuals showed a trend toward a higher frequency of HPyV infections. Interestingly, although not statistically significant, a higher proportion of subjects harboring multiple β-HPV infections was observed in the high-risk group (≥5: *p* = 0.52, OR = 0.25, 95% CI: 0.003, 6.02; ≥10: *p* = 0.25, OR = 0, 95% CI: 0, 3.97).

### 3.4. Distribution and Prevalence of Individual β-HPV Genotypes in Risk and Cancer Groups Compared to Controls

**Figure 3:**
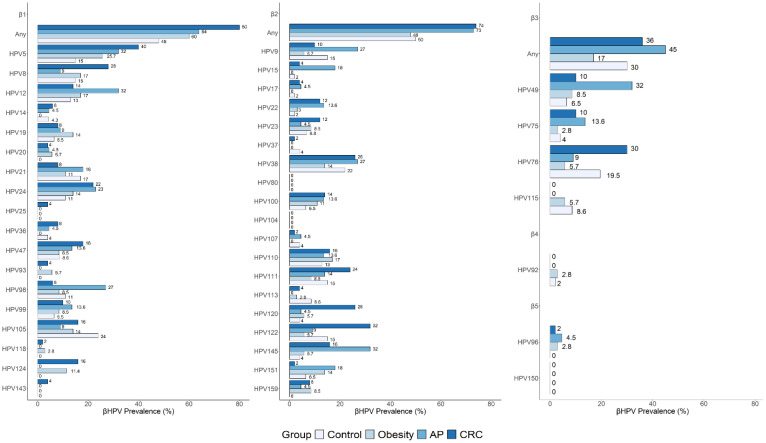
β-HPV species and individual genotypes in control, cancer, and risk groups. Species- and type-specific β-HPV distribution and prevalence in individuals with obesity (N = 35), AP (N = 22), and CRC (N = 50) are reported in comparison to the control group (N = 46).

Of the 45 β-HPV genotypes tested, 42 were detected across all study groups. Specifically, 34 genotypes were found in control individuals, 33 in both obesity and AP groups, and 40 in CRC patients. The distribution and prevalence of specific β-HPV species and individual genotypes in cancer and risk groups, and the comparisons with the control group, are reported in Figure 3 and Supplementary Table S1. HPV80 and HPV104 (β2 species) and HPV150 (β5) were not detected in any study group. As shown in Figure 3 and Supplementary Table S1, the species-specific prevalence in controls was 48% for β1, 50% for β2, 30% for β3, 2% for β4, and less than 2% for β5. The frequencies of individual genotypes ranged from less than 2% to a maximum of 24%, according to previously reported results [34]. The most frequently detected genotypes in this group included β-1 types HPV5, HPV8, HPV21, and HPV105; β-2 types HPV9, HPV38, HPV111, and HPV122; and the β-3 type HPV76 (Figure 3 and Supplementary Table S1).

A significantly higher prevalence of both β1 and β2 species was observed among CRC patients compared to controls (β1: 80% vs. 48%, *p* = 0.001; β2: 74% vs. 50%, *p* = 0.02), with a similar trend seen in the AP group (β1: 64% vs. 48%; *p* = 0.3; β2: 73% vs. 50%; *p* = 0.12). Several individual genotypes were more frequently detected in the cancer and risk groups compared to the control group (Figure 3 and Supplementary Table S1). Among those significantly increased, HPV5, HPV120, and HPV124 showed a higher prevalence in CRC patients, while HPV15, HPV49, and HPV145 were more prevalent in the AP group (Supplementary Table S1). Interestingly, HPV124 was also significantly more frequent in individuals with obesity. Conversely, some genotypes, such as HPV37, HPV92, HPV105, HPV113, HPV115, and HPV152, were either absent or less prevalent in disease groups compared to healthy controls. Over 30% of β-HPV types showed no variations across groups.

### 3.5. Overrepresented β-HPV Genotypes in Cancer and Risk Groups Versus Control Group: Unique and Shared Profiles

**Figure 4:**
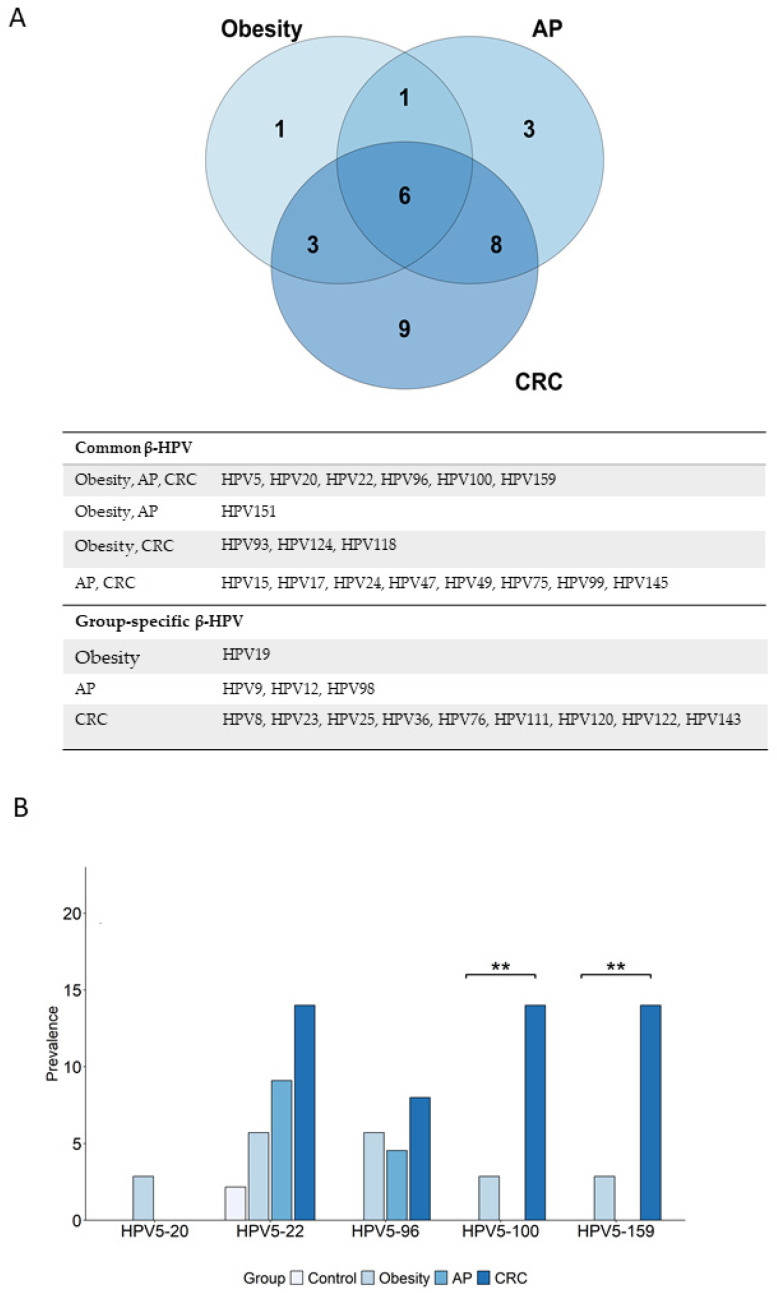
Overrepresented β-HPV genotypes in cancer and risk groups compared to controls. β-HPV genotypes overrepresented in the cancer (N = 50) and risk (obesity, N = 35; AP, N = 22) groups versus controls (N = 46) (at least a 1.5-fold increase in prevalence) are reported. (**A**) Venn diagram and table showing the number of genotypes specific to each indicated subject group or shared by groups. (**B**) Prevalence of co-infections of HPV5 with each indicated β-HPV genotype. Comparisons were performed using Fisher’s exact test, and statistical significance is indicated (** *p* < 0.01).

The increased prevalence of several β-HPV genotypes in the CRC and risk groups compared to controls suggests a potential role for these viruses as risk indicators. A Venn diagram was used to assess the number of overrepresented (showing at least a 1.5-fold increase in prevalence) viral genotypes in the cancer and risk groups versus controls, as well as their overlapping or uniqueness. As shown in Figure 4A, most of the overrepresented β-HPV genotypes (N = 26) were found in the CRC group, while 11 were overrepresented in the obesity group and 18 in the AP group. Nine genotypes were CRC-specific, three were specific to AP, and only one was obesity-specific. Additionally, the CRC group shared 14 genotypes with the AP group and 9 with the obesity group. Among these, six β-HPV types, HPV5, HPV20, HPV22, HPV96, HPV100, and HPV159, were common to all cancer and risk groups, suggesting a possible role in cancer risk. Of note, three out of the six common genotypes (HPV20, HPV96, and HPV159) were entirely absent in healthy controls (Figure 3). Interestingly, HPV5, classified by IARC as a “Group 2B carcinogen”, was among the genotypes shared across all cancer and risk groups (Figure 4A). We further analyzed the co-infection patterns of HPV5 with the other common genotypes. As shown in Figure 4B and Supplementary Table S2, HPV5 frequently co-occurred with HPV22, HPV96, HPV100, or HPV159 in both risk groups and particularly in the cancer group.

Focusing on the genotypes overrepresented in the CRC group or shared with one or both risk groups (Figure 4A), two cancer-relevant β-HPV categories were identified: (i) viruses with oncogenic potential (HPV5, HPV8, HPV76, and HPV122), and (ii) viruses previously detected in CRC tissues or adjacent intestinal mucosa [18], hereafter referred to as “CRC tissue-associated” (HPV20, HPV23, HPV24, HPV49, HPV93, HPV120, and HPV124).

### 3.6. Prevalence of Cancer-Relevant β-HPV Genotypes Is Increased in Risk and Cancer Groups, and in Healthy Controls According to BMI

**Figure 5:**
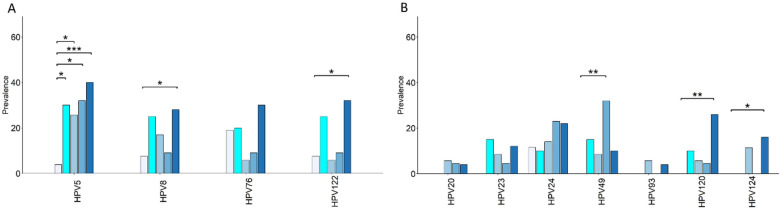
Prevalence of relevant β-HPV genotypes in all groups and impact of BMI in the control group. The prevalence of β-HPV genotypes with oncogenic potential (**A**) or CRC tissue-associated (**B**) is reported in the CRC (N = 50), obesity (N = 35), and AP (N = 22) groups, as well as in the overweight (OW, N = 20) and normal weight (NW, N = 26) control subgroups. Comparisons were performed using Fisher’s exact test, and statistical significance versus the NW control group is indicated (* *p* < 0.05, ** *p* < 0.01, *** *p* < 0.001).

Increased BMI is linked with chronic inflammation and higher susceptibility to infection. Moreover, OW individuals are at increased risk of CRC as compared to their NW counterparts [54]. The control group (N = 46) was thus subgrouped into age-matched NW (N = 26) and OW (N = 20) subjects to investigate whether β-HPV prevalence could differ depending on BMI. Focusing on β-HPV genotypes with oncogenic potential or “CRC tissue-associated” revealed differences according to BMI and disease status (Figure 5 and Table 3).

Most genotypes showed a higher prevalence in OW compared to the age-matched NW controls. Notably, the prevalence of HPV5 was significantly increased in all groups relative to the healthy NW controls (Figure 5A and Table 3). In addition, significantly higher prevalences of HPV8, HPV122, HPV120, and HPV124 were found in CRC patients compared to NW controls (Figure 5A,B and Table 3). Conversely, HPV49 prevalence was significantly increased in the AP group (Figure 5B). Interestingly, except for HPV24, genotypes previously found in CRC tissues (“CRC tissue-associated”) were absent in the oral cavity of healthy NW controls, and their prevalence increased in all cancer and risk groups, including OW individuals (Figure 5B).

### 3.7. Bariatric Surgery Shapes Oral Viral Profiles in Individuals with Obesity and Reduces the Prevalence of Cancer-Relevant β-HPV Infections

Several epidemiological studies have reported a significant decrease in CRC risk in subjects with obesity undergoing BS [55]. Given that oral virus profiles are altered in CRC patients, in the AP and obesity risk groups, and even in OW controls, we investigated whether a lifestyle intervention such as BS could modulate the prevalence of HPV, HPyV, and HHV infections. To this end, viral genotyping was performed on oral samples from subjects with obesity at baseline (T0) and 6 (T6) and 12 (T12) months post-surgery.

**Figure 6:**
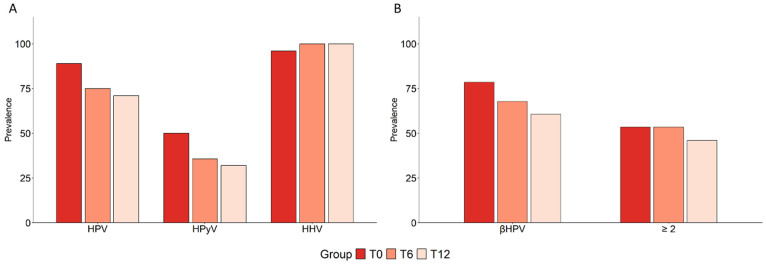
Effect of bariatric surgery on HPV, HPyV, HHV, and on β-HPV infections and multiple infections in subjects with obesity. (**A**) The prevalence of HPV, HPyV, and HHV infections found in the oral cavity of subjects with obesity (N = 28) before (T0) and six (T6) and twelve (T12) months after BS is shown. (**B**) The prevalence of β-HPV and multiple (≥2) β-HPV infections calculated at the same time points is shown. Comparisons were performed using Fisher’s exact test.

A reduction in HPV and HPyV prevalence was observed at both T6 and T12 compared to the baseline (Figure 6A and Table 4). The reduction in HPyV was primarily driven by a decrease in MCPyV. In contrast, no significant changes were observed in the distribution of HHV.

For β-HPV, a progressive reduction in overall prevalence was found post-surgery, with only a modest decrease in the frequency of multiple infections observed at T12 (Figure 6B and Table 4). Concurrently, inflammatory markers were analyzed in subjects with obesity who were positive for any β-HPV at baseline. As shown in Supplementary Figure S2, circulating levels of IL-6, TNF-α, and leptin were significantly reduced over time, in parallel with an increase in the levels of adiponectin. Additionally, a significant reduction in ccf-mtDNA, as a damage-associated molecular pattern, was observed after BS, suggesting that BS-induced weight loss and recovery of immune functions may contribute to viral clearance.

**Figure 7:**
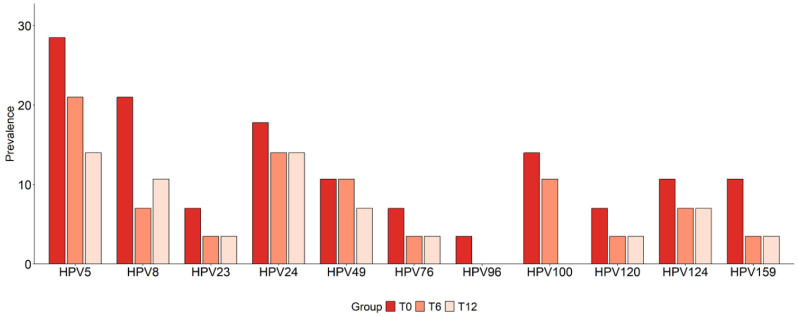
Effect of bariatric surgery on infections by individual β-HPV genotypes in subjects with obesity. The prevalence of infections by the indicated β-HPV genotypes in the oral cavity of subjects with obesity (N = 28), calculated at baseline (T0) and six (T6) and twelve (T12) months after bariatric surgery, is reported. Comparisons were performed using Fisher’s exact test.

We next examined whether the frequencies of the specific β-HPV genotypes were modulated by BS. This analysis revealed that, among 33 genotypes detected in the oral cavity of individuals with obesity at the baseline, 15 exhibited reduced prevalence at T6 and/or T12. Interestingly, this group included 11 β-HPV types we found to be associated with CRC risk (Figure 7). Specifically, reduced frequencies were observed for most of the β-HPV types: (i) identified as common to cancer and risk groups (HPV5, HPV96, HPV100, HPV159), (ii) CRC tissue-associated (HPV23, HPV24, HPV49, HPV120, HPV124), and (iii) with oncogenic potential (HPV5, HPV8, HPV76) (Figure 7 and Table 5).

Four additional β-HPV types (HPV19, HPV92, HPV105, HPV113) showed reduced prevalence post-surgery (Table 5). Conversely, the effect of BS on some virus types, selectively or more strongly overrepresented among AP patients, was not observed (HPV145, HPV12, HPV98) or was not analyzed (HPV15) due to the absence of this infection in the obesity cohort.

Confounding variables (smoking, age, and BMI) were tested to inspect their role in prevalence changes of these β-HPV genotypes across subject groups. Only age was found to be a confounding factor for two of the selected genotypes, HPV8 (standardized mean difference, SMD 0.72) and HPV120 (SMD 0.94), thus weakening their association with CRC risk.

### 3.8. Sex Differences in Oral β-HPV Genotype Profiles

**Figure 8:**
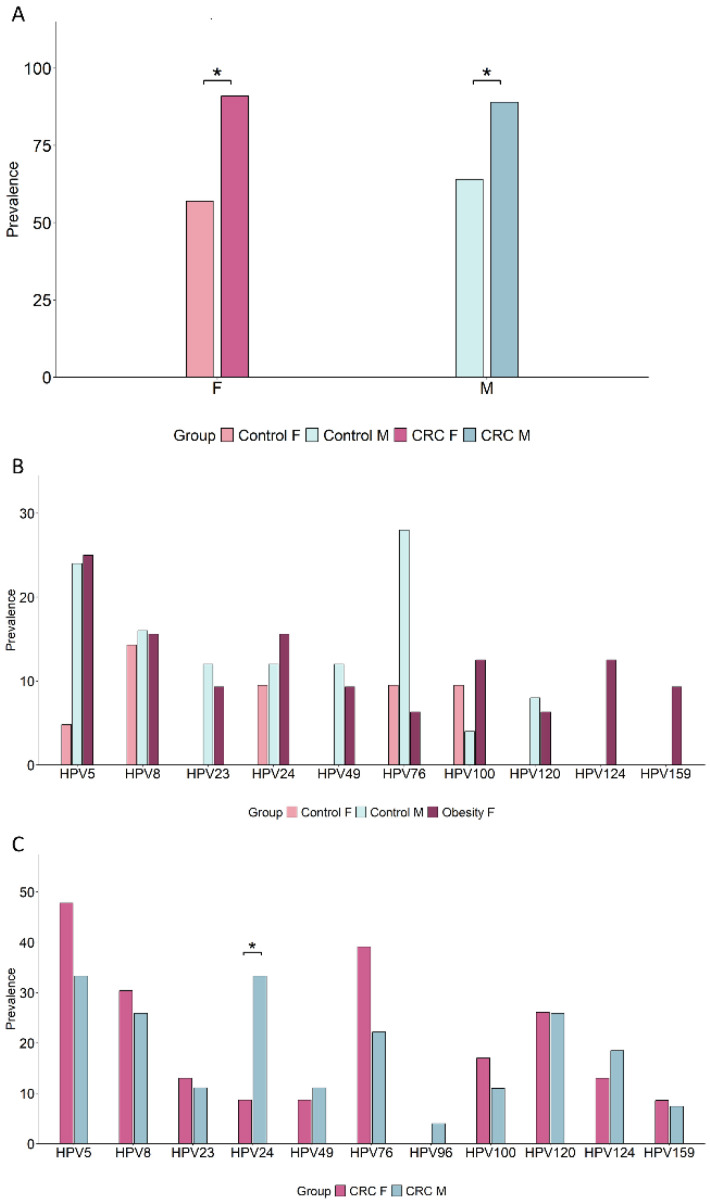
Sex differences in the prevalence of β-HPV and CRC risk-related β-HPV genotypes. Male and female subgroups within the control (25/21; (**A**,**B**)) and CRC (27/23; (**A**,**C**)) groups were compared for the prevalence of any β-HPV (**A**) or of the indicated β-HPV genotypes (**B**,**C**). The comparison of male and female control subgroups with the group of women with obesity (N = 32) is reported in (**B**) (* *p* < 0.05).

Sex-based disparities in CRC incidence have been documented, with men generally at higher risk than women [56]. To explore potential sex differences in the prevalence of β-HPV and of the selected β-HPV genotypes, we analyzed their sex-related distribution in the control and CRC groups, which had balanced male and female representation (Table 1). As shown in Figure 8A, no significant sex-related intra-group differences were observed in overall β-HPV prevalence. Moreover, the increase in β-HPV prevalence in CRC patients versus controls was statistically significant in both sexes. However, genotype-specific differences were noted in the control group [males/females 25/21, mean age 50.84/52.8 (*p* = 0.61), mean BMI 25.23/24.03 (*p* = 0.12)] (Figure 8B and Supplementary Table S3). Specifically, a trend toward a higher prevalence of HPV5, HPV23, HPV49, HPV76, and HPV120 was found in control men (SMD ≥ 0.8), while HPV100 (SMD 0.5) was more frequent in females, although these differences did not reach statistical significance. Conversely, in the CRC group, viral genotype frequencies were largely comparable between sexes, except for HPV24, which was significantly more prevalent in men (Figure 8C and Supplementary Table S3). To further investigate whether the lower prevalence of infections by specific β-HPV types in control women could be linked to a reduced CRC risk, we compared male and female controls to the age-matched group of women with obesity (N = 32, mean age 52.31, *p* = 0.89 versus control females, *p* = 0.53 versus control males). As shown in Figure 8B, in the risk group of women with severe obesity, the prevalence of infections by these genotypes was markedly higher with respect to control women, and comparable to that found in control men.

## 4. Discussion

In this study, we analyzed the prevalence of human DNA viruses in the oral cavity of CRC patients and subjects with cancer-predisposing conditions, assessing their role as potential risk indicators. While several previous studies reported the presence of viral DNA in CRC tissues [15,16,18], this is the first broad-spectrum analysis of the three major viral families, HPV, HPyV, and HHV, in the oral cavity of CRC-affected subjects. A comparative analysis was also performed in cohorts of cancer patients and subjects with AP or severe obesity, who have a higher risk of developing CRC compared to the healthy population. Despite the small sample size, our results highlighted a higher prevalence of the β-HPV genus and specific β-HPV genotypes in both CRC patients and risk groups compared to healthy controls, within a cross-sectional framework.

The prevalence of oral β-HPV infections in healthy populations varies widely across studies but rarely exceeds 60% even in older cohorts [30,33,34,35,36,39,40,57]. The prevalence in our control group aligns with several published studies [34,35,36,39,40], although it was higher than that reported in others [30,33,58,59]. Geographical, sex-specific, and lifestyle-related factors, including body weight and adiposity, may partially explain discrepancies across studies. Notably, the distribution of individuals with normal weight, overweight, or obesity in the study populations has never been considered in previous studies. Our results provide novel evidence of increased oral β-HPV prevalence in CRC patients and high-risk cohorts of subjects with precancerous lesions or severe obesity compared to the healthy population. In addition, some specific β-HPV genotypes we selected as possibly associated with CRC risk were found to be more prevalent in OW controls than in the age-matched NW counterparts, allowing us to hypothesize a role for these viruses as early non-invasive and lifestyle-modifiable indicators for CRC risk.

Importantly, in support of our hypothesis, BS-associated weight loss was accompanied by observed decreases in most of these relevant viral genotypes in varying proportions. Among the six β-HPV types identified as “common” to CRC and all risk groups, four (HPV5, HPV96, HPV100, and HPV159) showed clearance in a proportion of subjects 12 months after BS. Notably, HPV5 was classified as possibly carcinogenic to humans (IARC Group 2B carcinogen, PMID: 206906) and has been associated with oral and oropharyngeal cancers [40].

Additional potentially oncogenic β-HPV genotypes were also reduced after BS. Furthermore, BS was associated with observed clearance of infections by some β-HPV types (HPV23, HPV49, HPV120, and HPV124) previously identified in tumor tissue-adjacent colon mucosa of CRC patients [18] and here found overrepresented in the oral cavity of cancer and risk groups. Conversely, other “CRC tissue-associated” β-HPV types (i.e., HPV20, HPV24, HPV93) were not affected or marginally modulated by BS, raising questions about their riskiness. In conclusion, oral infections by 11 β-HPV genotypes, here selected as possible indicators of CRC risk, were cleared, in variable proportions of subjects, following a lifestyle intervention associated with reduced cancer risk, underscoring the modifiable nature of these indicators.

In this study, multiple oral β-HPV infections were also observed in control subjects, as reported in previous studies, and a marked increase in their frequency was reported in CRC patients and in subjects with high-risk colon polyps. Notably, the role of multiple HPV type co-infections in HPV-related cancers has been analyzed, showing an association with an increased risk of malignant transformation [60]. Furthermore, a significantly increased prevalence of co-infections of HPV with HPyV and HHV was also found in AP and CRC patients, suggesting a role in disease pathogenesis. In this regard, the lifelong interactions with HHV and HPyV types, sharing a similar epithelial niche, have been reported to enhance HPV persistence and accelerate the onset of HPV-induced genital cancers [61]. In this study, HHVs were found to be widely distributed in the oral cavity of healthy individuals. Moreover, apart from EBV1 overrepresentation in cancer patients, their prevalence was not substantially modulated in risk conditions and was not affected by BS. Conversely, some HPyV types showed increased prevalence in subjects with AP, although no association with polyp size and histology was found. Future studies on HPV co-infections with other potentially pathogenic viruses in the context of the whole oral microbiome are needed to better define their contribution to oral and gastrointestinal cancers.

Few studies have investigated the immune, hormonal, and lifestyle factors potentially influencing virus persistence in the oral cavity [34,37,62]. Chronic α-HPV infection has recently been associated with oral inflammation [48]. Likewise, chronic low-grade inflammation, oxidative imbalance, and immune dysregulation characterizing high CRC risk conditions, such as overweight, obesity, and AP, could contribute to virus immune escape and persistence [63]. Excessive immune cell activation, dysregulation of the antiviral type I IFN system, and TLR overstimulation by endogenous ligands, such as ccf-mtDNA, reported in these conditions, have been postulated as the fuel of inflammation and a driver of diseases, including cancer [64,65]. This could further result in impaired TLR recognition of exogenous virus-derived ligands, leading to immune evasion and persistent infections. In line with this hypothesis, we report a reduction in the prevalence of β-HPV type infections in subjects with obesity following BS, concomitantly with the reduction in body weight, systemic inflammatory markers, and ccf-mtDNA [50,66]. Although a causal link between the inflammatory status and β-HPV persistence could not be demonstrated, it could be hypothesized that in some subjects, the shutdown of inflammation following weight loss and the improved dietary habits, likely accompanied by a rescue of the immune function, may have led to the clearance of infections. This would suggest that these infections may contribute to the cancer-promoting effects of pro-inflammatory diets or unhealthy lifestyles and may also help monitor the effectiveness of primary prevention strategies. Accordingly, a reduction in persistent genital α-HPV infections was reported following dietary supplementation with antioxidant and immunostimulatory fungal mycelia extracts, which was associated with an improved immune response [67].

Persistent viral infections may also be maintained by virus–bacteria interactions within the oral microbiome. In this regard, an increased *Streptococcus sanguinis* abundance has been associated with EBV reactivation [68]. In turn, chronic viral infections and multiple infections can sustain inflammation and immunosuppression, creating a permissive environment for carcinogenesis [69]. Changes in both gut and oral bacterial microbiomes have been described in obesity as well as in individuals with precancerous colon lesions or CRC [70,71,72]. Furthermore, gut translocation of some oral pathobiont bacteria has been linked to intestinal carcinogenesis [73]. Although not yet described, oral-to-gut translocation of viruses may also occur, contributing, in synergy with other risk factors, to CRC development. The observation that several β-HPV genotypes, here found overrepresented in the oral cavity, were also detected in colon mucosa and cancer tissues [18] would support this hypothesis, which remains speculative and requires verification in prospective studies. This cross-sectional study only reflects adjusted associations and cannot establish causality, mechanisms, or prognostic value.

Sex differences have been described in CRC incidence, with men being at a higher risk of developing cancer compared to women [56]. In this regard, a higher oral β-HPV prevalence has been reported for men compared to women in studies involving a healthy population from Hong Kong [59,62]. However, the role of sex has not always been correctly investigated, as most studies analyzed men or women separately [30,33,38,39]. In our study, overall β-HPV prevalence in the healthy control group did not differ significantly between sexes. However, among the β-HPV genotypes we identified as modifiable CRC risk indicators, and/or potential cofactors in cancer pathogenesis, HPV5, HPV23, HPV49, HPV76, and HPV120 were more frequently detected in control men as compared to the age- and BMI-matched women subgroup. Notably, in the age-matched group of women with severe obesity, who have a higher cancer risk, β-HPV genotype prevalence was comparable to that of control men, suggesting that inflammatory or immunocompromising conditions may diminish women’s protective advantage. Based on several studies highlighting sex differences in immune response [74], this finding would suggest that healthy women may have better immune control over oral virus infections as well as cancer onset compared to men, and that this protection is lost in conditions of chronic inflammation and immunosuppression. Due to the observational nature and the small size of this study, which has not always allowed for achieving statistical significance, the sex-specific contribution of these viruses to cancer risk and pathogenesis deserves further investigation.

The increased prevalence of specific oral β-HPV types we reported in CRC patients and high-risk subject groups takes on further relevance as some of these viral genotypes have been shown to have transforming properties in in vitro and in vivo experimental models [75,76], and to play a role in non-melanoma skin cancers and head and neck cancers [36,40]. This would suggest that these subject groups could be exposed to the risk of developing other neoplastic diseases in addition to CRC. In this regard, abdominal obesity has been associated with an increased risk of head and neck cancer [77]. Our results reinforce the concept of the oral cavity as a viral reservoir, with increased HPV prevalence in immunocompromised states, underscoring the need for further research into their oncogenic potential. Currently, there are no screening or prevention guidelines for β-HPV infections in the oral cavity. However, β-HPV types have been included in the list of recommended priorities for carcinogenicity evaluations by the International Agency for Research on Cancer (IARC) Monographs program 2025–2029 [78].

## 5. Conclusions

CRC is a major global health issue, with increasing incidence rates in individuals younger than 50. Anticipating the detection of early, curable disease stages has the potential to reduce the health burden. Furthermore, the early detection of modifiable risk factors is critical. In this pilot study, we identified a panel of oral β-HPV genotypes that may serve as potential early non-invasive indicators of CRC risk. The sex differences in their frequencies and their modifiable nature could allow the identification of high-risk individuals who would benefit from lifestyle interventions and could help guide prevention towards sex-oriented strategies. Future prospective studies with larger and sex-stratified cohorts are required to validate the risk-predictive value of these viral types and to assess their utility in clinical practice and public health interventions.

## Figures and Tables

**Table 1:** Anthropometric and clinical characteristics of the study populations.

Variables	Control (N = 46)	Obesity (N = 35)	AP (N = 22)	CRC (N = 50)
***Age*, years, mean (SD)**	51.7 (12.44)	52.6 (10.84)	65.4 (11.6)	70.6 (8.98)
				
***Sex, n (%)***				
Female	21 (45.7)	32 (91.4)	5 (22.7)	23 (46)
Male	25 (54.3)	3 (8.6)	17 (77.3)	27 (54)
				
***BMI*, kg/m^2^, mean (SD)**	24.68 (2.7)	44.75 (6.49)	26.15 (3.46)	25.55 (3.6)
				
***Tumor stage, n (%)***				
0–I	-	-	-	12 (24)
II	-	-	-	25 (50)
III–IV	-	-	-	13 (26)
				
***Polyp features, n (%)***				
***Number***				
Single	-	-	14 (63.6)	-
Multiple (2–4)	-	-	8 (36.4)	-
*Size*				
≤10 mm	-		5 (23)	
>10 mm	-	-	17 (77)	-
*Villous histology*	-		7 (31.8)	
*Dysplasia degree*				
Low grade	-	-	20 (91)	-
High grade	-	-	2 (9)	-
				
***Smoker, n (%)***				
Never	29 (66)	20 (57)	13 (59)	21 (42)
Former	7 (16)	9 (25.7)	4 (18)	21 (42)
Current	8 (18)	6 (17)	5 (23)	8 (16)

**Table 2:** Prevalence of oral HPV, HPyV, and HHV families and individual HPyV and HHV genotypes in the study populations. Virus prevalence in subjects with obesity or AP and in CRC patients is shown compared to the control group. The odds ratio (OR) values along with 95% confidence intervals (CIs) and *p*-values are reported. Comparisons were performed using Fisher’s exact test, and the statistically significant (*p* < 0.05) results are highlighted in bold.

Family	Genus/Genotype	Control	Obesity			AP			CRC		
		*%*	*%*	*OR* *(95% CI)*	*p*	*%*	*OR* *(95% CI)*	*p*	*%*	*OR* *(95% CI)*	*p*
HPV	Any	72	86	2.34 (0.68–9.41)	0.18	77	1.33 (0.36–5.60)	**0.77**	92	4.46 (1.23–20.47)	**0.01**
	α	9	8	0.97 (0.13–6.27)	1	0	0 (0–3.15)	0.30	10	1.16 (0.23–6.28)	1
	β	61	77	2.15 (0.74–6.73)	0.15	73	1.7 (0.51–6.34)	0.42	90	5.68 (1.77–21.82)	**0.001**
	γ	41	31	0.65 (0.23–1.80)	0.49	45	1.18 (0.37–3.71)	0.80	44	1.11 (0.46–2.71)	0.84
HPyV	Any	41	43	1.06 (0.40–2.84)	1	73	3.71 (1.12–13.85)	**0.02**	60	2.11 (0.87–5.22)	0.10
	HPyV6	4.34	9.37	2.25 (0.24–28.50)	0.40	22.70	6.27 (0.92–71.93)	**0.03**	4	0.92 (0.06–13.15)	1
	MCPyV	41.30	40.62	0.97 (0.35–2.67)	1	54.50	1.69 (0.54–5.39)	0.43	62	1.95 (0.81–4.79)	0.15
HHV	Any	96	97	1.54 (0.08–93.77)	1	100	Inf (0.09-Inf)	1	96	1.09 (0.08–15.63)	1
	EBV1	34.78	40.62	1.28 (0.45–3.59)	0.64	40.90	1.29 (0.39–4.14)	0.79	64	3.29 (1.33–8.40)	**0.007**
	EBV2	4.34	3.12	0.71 (0.01–14.25)	1	13.63	3.40 (0.36–43.83)	0.32	0	0 (0–4.88)	0.23
	HSV1	6.52	18.75	3.25 (0.63–21.84)	0.15	13.63	2.23 (0.27–18.25)	0.38	10	0 (0–4.88)	0.23
	CMV	2.17	9.37	4.56 (0.35–249.33)	0.30	4.54	2.12 (0.03–3.78)	0.37	6	1.86 (0.09–112.96)	1
	HHV6B	41.30	62.62	2.45 (0.89–7.06)	0.07	68.18	2.74 (0.85–9.58)	0.07	46	1.02 (0.42–2.47)	1
	HHV7	95.65	87.50	0.44 (0.03–4.13)	0.40	90.90	0.46 (0.03–6.77)	0.59	88	0.41 (0.04–2.68)	0.44

OR, odds ratio; CI, confidence interval; Inf., infinite.

**Table 3:** Prevalence of relevant β-HPV genotypes in all groups and impact of BMI in healthy controls. The prevalence of individual β-HPV genotypes in the overweight (OW, N = 20) control subgroup and in subjects with obesity (N = 35), AP (N = 22), or CRC (N = 50) is shown compared to the normal weight (NW, N = 26) control subgroup. The odds ratio (OR) values along with 95% confidence intervals (CIs) and *p*-values are reported. The absence of infections in the NW control group did not allow calculation of OR values. Comparisons were performed using Fisher’s exact test, and the statistically significant (*p* < 0.05) results are highlighted in bold.

β-HPV Type	NW	OW			Obesity			AP			CRC		
	*%*	*%*	*OR* *(95% CI)*	*p*	*%*	*OR* *(95% CI)*	*p*	*%*	*OR* *(95% CI)*	*p*	*%*	*OR* *(95% CI)*	*p*
HPV5	3.8	30	10.2 (1.07–10.48)	**0.03**	25.7	8.41 (1.03–393.37)	**0.03**	32	11.11 (1.24–544.58)	**0.02**	40	16.21 (2.27–715.30)	**0.0008**
HPV8	7.6	25	3.88 (0.55–5.65)	0.21	17	2.4 (0.39–26.98)	0.45	9	1.19 (0.08–7.88)	1	28	4.59 (0.92–45.23)	**0.04**
HPV20	0	0	0 (/)	1	5.7	/	0.50	4.5	/	0.46	4	/	0.54
HPV23	0	15	/	0.07	8.5	/	0.25	6.5	/	0.46	12	/	0.09
HPV24	11.5	10	0.85 (0.06–8.31)	1	14	1.27 (0.22–9.05)	1	11	2.16 (0.37–6.28)	0.44	22	2.14 (0.49–13.19)	0.36
HPV49	0	15	0 (/)	0.07	8.5	/ (/)	0.25	6.5	/	**0.002**	10	0.49 (/)	0.16
HPV76	19	20	1.05 (0.18–5.78)	1	5.7	0.26 (0.02–1.77)	0.12	9	0.43 (0.04–2.99)	0.43	30	1.79 (0.52–7.22)	0.41
HPV93	0	0	0 (/)	1	5.7	/	0.50	0	/	1	4	/	0.54
HPV120	0	10	/	0.18	5.7	/	0.50	4.5	/	0.46	26	/	**0.003**
HPV122	7.6	25	3.88 (0.55–5.65)	0.21	5.7	0.73 (0.05–0.75)	1	9	1.19 (0.08–17.88)	1	32	5.54 (1.13–54.12)	**0.02**
HPV124	0	0	0 (/)	1	11.4	/	0.1	0	/	1	16	/	**0.04**

OR, odds ratio; CI, confidence interval; NW, normal weight; OW, overweight; /, not applicable.

**Table 4:** Oral virus prevalence in subjects with obesity after bariatric surgery. The prevalence of HPyV, HPV, β-HPV, and multiple β-HPV infections (≥2) found in the oral cavity of subjects with obesity before (T0) and six (T6) and twelve (T12) months after BS is shown. The odds ratio (OR) values along with 95% confidence intervals (CIs) and *p*-values are reported. Comparisons were performed using Fisher’s exact test.

Virus	%			T6 vs. T0		T12 vs. T0		T12 vs. T6	
	*T0*	*T6*	*T12*	*OR (95% CI)*	*p*	*OR (95% CI)*	*p*	*OR (95% CI)*	*p*
**HPyV**	50	35.7	32	0.56 (0.16–1.84)	0.42	0.48 (0.14–1.59)	0.28	0.85 (0.24–2.97)	1
**HPyV6**	9.1	3	6.9	0.31 (0.005–4.21)	0.61	0.65 (0.05–6.14)	1	2.05 (0.10–126.89)	1
**MCPyV**	42.4	39.4	31	0.88 (0.28–2.69)	1	0.70 (0.21–2.26)	0.59	0.79 (0.23–2.60)	0.79
**HPV**	89	75	71	0.58 (0.14–2.23)	0.55	0.43 (0.11–1.57)	0.25	0.74 (0.21–2.51)	0.78
**β-HPV**	78.5	67.8	60.7	0.58 (0.14–2.23)	0.55	0.43 (0.11–1.57)	0.25	0.74 (0.21–2.51)	0.78
**Multiple** **infections**	53.5	53.5	46	1 (0.31–3.24)	1	0.75 (0.23–2.43)	0.79	0.75 (0.23–2.43)	0.79

OR, odds ratio; CI, confidence interval.

**Table 5:** Effect of bariatric surgery on the prevalence of individual β-HPV genotypes. The prevalence of individual β-HPV genotypes found in the oral cavity of subjects with obesity before (T0) and six (T6) and twelve (T12) months after BS is shown. The odds ratio (OR) values along with 95% confidence intervals (CIs) and *p*-values are reported. Statistical comparisons were performed using Fisher’s exact test.

Genotype	%			T6 vs. T0		T12 vs. T0		T12 vs. T6	
	*T0*	*T6*	*T12*	*OR* *(95% CI)*	*p*	*OR* *(95% CI)*	*p*	*OR* *(95% CI)*	*p*
**HPV5**	28.5	21	14	0.69 (0.16–2.72)	0.76	0.42 (0.08–1.87)	0.33	0.62 (0.11–3)	0.73
**HPV8**	21	7	10.7	0.29 (0.02–1.82)	0.25	0.45 (0.64–2.39)	0.47	1.55 (0.16–19.99)	1
**HPV19**	17.8	14	10.7	0.77 (0.13–4.08)	1	0.56 (0.078–3.24)	0.70	0.72 (0.096–4.78)	1
**HPV23**	7	3.5	3.5	0.49 (0.008–9.90	1	0.49 (0.008–9.90)	1	1 (0.12–81.32)	1
**HPV24**	17.8	14	14	0.77 (0.13–4.08)	1	0.77 (0.13–4.08)	1	1 (0.16–6.04)	1
**HPV49**	10.7	10.7	7	1 (0.12–8.21)	1	0.65 (0.05–6.14)	1	0.65 (0.05–6.14)	1
**HPV76**	7	3.5	3.5	0.49 (0.008–9.9)	1	0.49 (0.008–9.9)	1	1 (0.12–81.32)	1
**HPV92**	3.5	0	0	0 (0–39)	1	0 (0–39)	1	0 (/)	1
**HPV96**	3.5	0	0	0 (0–39)	1	0 (0–39)	1	0 (/)	1
**HPV100**	14	10.7	0	0.72 (0.095–4.78)	1	0 (0–1.45)	0.11	0 (0–2.37)	0.24
**HPV105**	14	7	7	0.47 (0.04–3.61)	0.67	0.47 (0.04–3.61)	0.67	1 (0.68–14.76)	1
**HPV113**	3.5	0	0	0 (0–39)	1	0 (0–39)	1	0 (/)	1
**HPV120**	7	3.5	3.5	0.48 (0.008–9.90)	1	0.48 (0.008–9.90)	1	1 (0.12–81.32)	1
**HPV124**	10.7	7	7	0.65 (0.5–6.14)	1	0.65 (0.5–6.14)	1	1 (0.68–14.76)	1
**HPV159**	10.7	3.5	3.5	0.31 (0.006–4.21)	0.61	0.31 (0.006–4.21)	0.61	1 (0.012–81.32)	1

OR, odds ratio; CI, confidence interval; /, not applicable.

## Data Availability

All relevant data that support the findings of this study are included in the manuscript and are available from the corresponding author upon reasonable request.

## Supplementary Materials

The following supporting information can be downloaded at https://www.mdpi.com/article/10.3390/cancers17183024/s1, Supplementary Data S1–S5; Supplementary Figure S1: Effect of tumor stages and polyp features on virus infections; Supplementary Table S1: Prevalence of individual β-HPV genotypes in the study populations; Supplementary Figure S2: Analysis of inflammation markers in β-HPV-positive individuals with obesity, before and after BS; Supplementary Table S2: Prevalence of co-infections of HPV5 with each indicated β-HPV genotype in the study population; Supplementary Table S3: Prevalence of β-HPV genotypes in control and CRC male and female subjects.
