# Supplementary material for: Oral Viral DNA Profiling in Obesity, Adenomatous Polyposis, and Colorectal Cancer Identifies Human β-Papillomavirus Types as Potentially Sex-Related and Modifiable Cancer Risk Indicators
Source: Cancers (Basel). 2025 Sep 16;17(18):3024. doi: 10.3390/cancers17183024 (PMC12468992; doi:10.3390/cancers17183024)
Supplement: Supplementary file 1 [file cancers-17-03024-s001.zip › Supplemental Figure S1 .pdf]

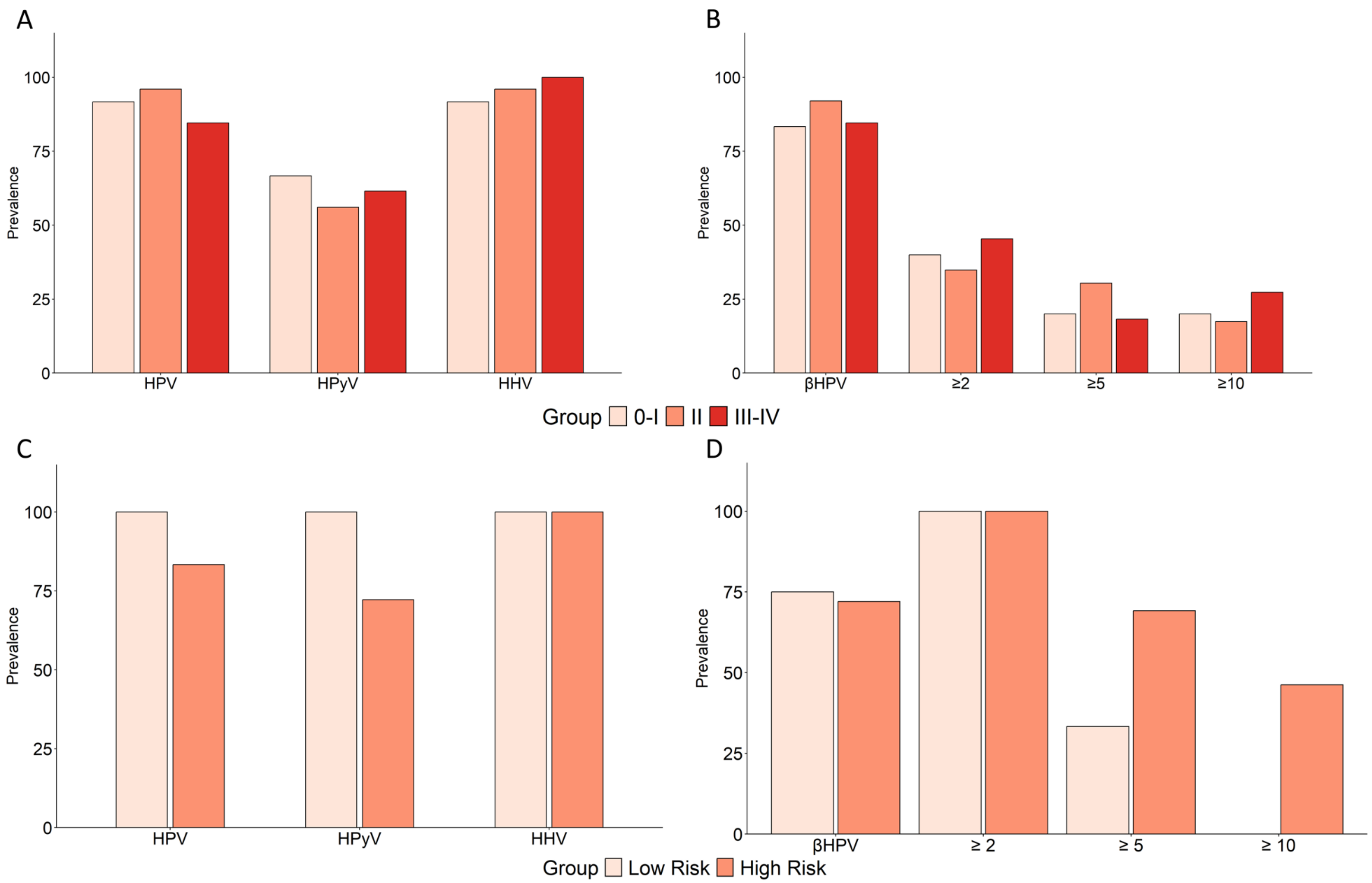
A
B
C
D
Prevalence
0
25
50
75
100
HPV
HPyV
HHV
βHPV
≥2
≥5
≥10
Group 0-I II III-IV
≥ 2
≥ 5
≥ 10
Group Low Risk High Risk

Supplemental Figure S1. Effect of tumor stages and polyp features on virus infections. The prevalence of HPV, HPyV and HHV (A, C) and of β-HPV infections and multiple infections (B, D) is shown for CRC patients (A, B) and subjects with AP (C, D) at the indicated tumor stages or polyp severity. Tumor stage was defined according to the TNM classification; high-risk polyps were defined as showing high-grade dysplasia, size grater than 10 mm or villous histology. Comparisons were performed using Fisher exact test.
