# Supplementary material for: Oral Viral DNA Profiling in Obesity, Adenomatous Polyposis, and Colorectal Cancer Identifies Human β-Papillomavirus Types as Potentially Sex-Related and Modifiable Cancer Risk Indicators
Source: Cancers (Basel). 2025 Sep 16;17(18):3024. doi: 10.3390/cancers17183024 (PMC12468992; doi:10.3390/cancers17183024)
Supplement: Supplementary file 1 [file cancers-17-03024-s001.zip › Supplemental Table S1 .pdf]

Supplemental Table S1. Prevalence of individual β-HPV genotypes in the study population.

Genotype	Control	Obesity			AP			CRC		
	%	*%*	*p*	*OR (95% CI)*	*%*	*p*	*OR (95% CI)*	*%*	*p*	*OR (95% CI)*
**β1**										
**Any**	48	60	0,37	1.63 (0.61-4.39)	64	0,30	1.89 (0.60-6.29)	80	**0,001**	4.29 (1.63-12.04)
**HPV5**	15	25,7	0,27	1.91 (0.55-6.88)	32	0,20	2.56 (0.65-10.26)	40	**0,01**	3.66 (1.28-11.67)
**HPV8**	15	17	1	1.15 (0.29-4.48)	9	0,71	0.56 (0.05-3.33)	28	0,15	2.15 (0.71-7.05)
**HPV12**	13	17	0,75	1.37 (0.33-5.72)	32	0,10	3.05 (0.74-13.03)	14	1	1.08 (0.28-4.28)
**HPV14**	4,3	0	0,50	0 (0-6.99)	4,5	1	1.05 (0.17-21.18)	6	1	1.40 (0.15-17.49)
**HPV19**	6,5	14	0,28	2.36 (0.42-16.38)	9	0,65	1.42 (0.11-13.49)	8	1	1.24 (0.20-8.98)
**HPV20**	0	5,7	0,18	Inf (0.25-Inf)	4,5	0,32	Inf (0.05-Inf)	4	0,50	Inf (0.17-Inf)
**HPV21**	17	11	0,53	0.62 (0.12-2.57)	18	1	1.05 (0.20-4.60)	8	0,22	0.42 (0.08-1.70)
**HPV24**	11	14	0,74	1.36 (0.28-6.50)	23	0,27	2.38 (0.48-11.84)	22	0,17	2.29 (0.66-9.22)
**HPV25**	0	0	1	0 (0-Inf)	0	1	0 (0-Inf)	4	0,50	Inf (0.17-Inf)
**HPV36**	4	0	0,50	0 (0-7)	4,5	1	1.05 (0.02-21.18)	8	0,68	1.90 (0.26-22)
**HPV47**	8.6	8,5	1	0.98 (0.13-6.27)	13,6	0,67	1.64 (0.22-10.80)	18	0,24	2.28 (0.58-10.97)
**HPV93**	0	5,7	0,18	Inf (0.25-Inf)	0	1	0 (0-Inf)	4	0,50	Inf (0.17-Inf)
**HPV98**	11	8,5	1	0.77 (0.11-4.31)	27	0.16	3.02 (0.66-14.47)	6	0.48	0.53 (0.08-2.90)
**HPV99**	6,5	8,5	1	1.34 (0.17-10.66)	13,6	0,38	2.23 (0.27-18.25)	10	0,72	1.58 (0.29-10.83)
**HPV105**	24	14	0,40	0.53 (0.13-1.90)	9	0,20	0.32 (0.03-1.71)	16	0,44	0.61 (0.19-1.87)
**HPV118**	0	2,8	0,43	Inf (0.33-Inf)	0	1	0 (0-Inf)	2	1	Inf (0.02-Inf)

**HPV124**	0	11,4	**0,03**	Inf (0.90-Inf)	0	1	0 (0-Inf)	16	**0,006**	Inf (1.72-Inf)
**HPV143**	0	0	1	0 (0-Inf)	0	1	0 (0-Inf)	4	0,50	Inf (0.17-Inf)
**HPV152**	11	0	0,07	0 (0-1.38)	9	1	0.82 (0.07-5.58)	4	0,25	0.34 (0.03-2.25)
**β2**										
**Any**	50	48	1	0.94 (0.36-2.49)	73	0,12	2.63 (0.80-9.74)	74	**0,02**	2.81 (1.11-7.36)
**HPV9**	15	5,7	0,29	0.34 (0.03-1.96)	27	0,32	2.06 (0.49-8.50)	10	0,54	0.62 (0.14-2.49)
**HPV15**	2	0	1	0 (0-51.22	18	**0,03**	9.63 (0.88-501.54)	4	1	1.86 (0.09-112.96)
**HPV17**	2	0	1	0 (0-51.22)	4,5	0,55	2.12 (0.03-171.63)	4	1	1.86 (0.09-112.96)
**HPV22**	2	3	1	1.32 (0.02-106.08)	13,6	0,10	6.88 (0.51-380.14)	12	0,11	6.04 (0.69-287.99)
**HPV23**	6,84	8,5	1	1.34 (0.17-10.66)	4,5	1	0.69 (0.12-9.15)	12	0,49	1.94 (0.38-12.76)
**HPV37**	4	0	0,50	0 (0-6.99)	0	1	0 (9-11.21)	2	0,61	0.45 (0.007-8.97)
**HPV38**	22	14	0,56	0.60 (0.14-2.20)	27	0,76	1.34 (0.34-4.96)	26	0,64	1.26 (0.44-3.66)
**HPV80**	0	0	/	/	0	/	/	0	/	/
**HPV100**	6,5	11	0,46	1.83 (0.29-13.44)	13,6	0,38	2.23 (0.27-18.25)	14	0,32	2.31 (0.49-14.78)
**HPV104**	0	0	/	/	0	/	/	0	/	/
**HPV107**	4	0	0,50	0 (0-6.99)	4,5	1	1.05 (0.17-21.18)	2	0,61	0.45 (0.007-8.97)
**HPV110**	13	17	0,75	1.37 (0.33-5.72)	13,6	1	1.05 (0.15-5.59)	16	0,78	1.27 (0.35-4.85)
**HPV111**	15	8,5	0,50	0.53 (0.08-2.54)	14	1	0.88 (0.13-4.42)	24	0,31	1.75 (0.56-5.84)
**HPV113**	8,6	2,8	0,38	0.31 (0.006-3.35)	0	0,30	0 (0-3.15)	4	0,42	0.44 (0.04-3.26)
**HPV120**	4	5,7	1	1.33 (0.09-19.21)	4,5	1	1.05 (0.02-21.18)	26	**0,004**	7.58 (1.56-73.40)
**HPV122**	15	5,7	0,29	0.34 (0.03-1.96)	9	0,71	0.56 (0.05-3.33)	32	0,06	2.59 (0.88-8.40)
**HPV145**	4	5,7	1	1.33	32	**0,004**	9.85	16	0,09	4.13

				(0.09-19.21)			(1.64-107.38)			(0.76-42.22)
**HPV151**	6,5	14	0,28	2.36 (0.42-16.38)	18	0,20	3.12 (0.48-23.54)	2	0,35	0.30 (0.005-3.84)
**HPV159**	0	8,5	0,08	Inf (0.55-Inf)	4,5	0,32	Inf (0.05-Inf)	8	0,12	Inf (0.62-Inf)
**β3**										
**Any**	30	17	0,20	0.48 (0.13-1.54)	45	0,28	1.89 (0.58-6.13)	36	0,67	1.28 (0.50-3.31)
**HPV49**	6,5	8,5	1	1.34 (0.17-10.67)	32	**0,01**	6.47 (1.28-43.79)	10	0,72	1.58 (0.29-10.83)
**HPV75**	4	2,8	1	0.65 (0.01-12.98)	13,6	0,32	3.40 (0.36-43.83)	10	0,44	2.42 (0.37-26.72)
**HPV76**	19,5	5,7	0,10	0.25 (0.25-1.35)	9	0,48	0.41 (0.04-2.30)	30	0,35	1.75 (0.62-5,17)
**HPV115**	8,6	5,7	0,69	0.64 (0.05-4.78)	0	0,30	0 (0-3.15)	0	**0,05**	0 (0-1.35)
**β4**										
**HPV92**	2	2,8	1	1.32 (0.016-106.08)	0	1	0 (0-81.44)	0	0,48	0 (0-35.9)
**β5**										
**Any**	0	2,8	0,43	Inf (0.033-Inf)	4,5	0,32	Inf (0.053-Inf)	2	1	Inf (0.023-Inf)
**HPV96**	0	2,8	0,43	Inf (0.33-Inf)	4,5	0,32	Inf (0.54-Inf)	2	1	Inf (0.23-Inf)
**HPV150**	0	0	/	/	0	/	/	0	/	/

OR, odds ratio; CI, confidence interval; Inf., infinite; /, not applicable.
