# Supplementary material for: Oral Viral DNA Profiling in Obesity, Adenomatous Polyposis, and Colorectal Cancer Identifies Human β-Papillomavirus Types as Potentially Sex-Related and Modifiable Cancer Risk Indicators
Source: Cancers (Basel). 2025 Sep 16;17(18):3024. doi: 10.3390/cancers17183024 (PMC12468992; doi:10.3390/cancers17183024)
Supplement: Supplementary file 1 [file cancers-17-03024-s001.zip › Supplemental Table S2.pdf]

Supplemental Table S2. Prevalence of co-infections of HPV5 with each indicated β-HPV genotype in the study population.

Genotype	Control	Obesity			AP			CRC		
	%	*%*	*p*	*OR (95% CI)*	*%*	*p*	*OR (95% CI)*	*%*	*p*	*OR (95% CI)*
**HPV5-20**	0	2.86	0.43	Inf (0.03-Inf)	0	1	0 (0-61.99)	0	0	0 (0-Inf)
**HPV5-22**	2.17	5.71	0.57	2.69 (0.13-164.24)	9.1	0.24	4.39 (0.22-270.74)	14	0.06	7.20 (0.87-336.78)
**HPV5-96**	0	5.71	0.18	Inf (0.25-Inf)	4.54	0.32	Inf (0.05-16.05)	8	0.12	Inf (0.62-Inf)
**HPV5-100**	0	2.86	0.43	Inf (0.03-Inf)	0	1	0 (0-Inf)	14	**0.013**	Inf (1.43-Inf)
**HPV5-159**	0	2.86	0.43	Inf (0.03-Inf)	0	1	0 (0-Inf)	14	**0.013**	Inf (1.43-Inf)

OR, odds ratio; CI, confidence interval; Inf., infinite.
