# Supplementary material for: Oral Viral DNA Profiling in Obesity, Adenomatous Polyposis, and Colorectal Cancer Identifies Human β-Papillomavirus Types as Potentially Sex-Related and Modifiable Cancer Risk Indicators
Source: Cancers (Basel). 2025 Sep 16;17(18):3024. doi: 10.3390/cancers17183024 (PMC12468992; doi:10.3390/cancers17183024)
Supplement: Supplementary file 1 [file cancers-17-03024-s001.zip › Supplemental Table S3.pdf]

Supplemental Table S3. Prevalence of β-HPV genotypes in control and CRC male and female subjects.

Genotype	Control				CRC			
	% M	*% F*	*OR (95% CI)*	*p*	*% M*	*% F*	*OR (95% CI)*	*p*
**HPV5**	24	4.6	6.1 (0.65-304.23)	0.11	33.3	47.8	0.55 (0.15-1.98)	0.39
**HPV8**	16	14.3	1.14 (0.17-8.83)	1	25.9	30.4	0.8 (0.19-3.31)	0.76
**HPV23**	12	0	Inf (0.35-Inf)	0.24	11.1	13	0.84 (0.1-6.95)	1
**HPV24**	12	9.5	1.29 (0.13-16.95)	1	33.3	8,7	5.09 (0.89-54.38)	**0.04**
**HPV49**	12	0	Inf (0.35-Inf)	0.24	11.1	8.7	1.3 (0.13-17.03)	1
**HPV76**	28	9.5	3.60 (0.58-39.99)	0.15	22.2	39.1	0.33 (0.07-1.35)	0.11
**HPV96**	0	0	-	-	4	0	Inf (0.02-Inf)	1
**HPV100**	4	9.5	0.53 (0.00618.31)	0.58	11	17	0.6 (0.08-4.02)	0.59
**HPV120**	8	0	Inf (0.16-Inf)	0.49	25.9	26.1	1.50 (0.23-4.34)	1
**HPV124**	0	0	-	-	18.5	13	1.50 (0.25-10.93)	0.71
**HPV159**	0	0	-	-	7.4	8.6	0.84 (0.06-12.56)	1

OR, odds ratio; CI, confidence interval; Inf., infinite.
