# Supplementary material for: Oral Viral DNA Profiling in Obesity, Adenomatous Polyposis, and Colorectal Cancer Identifies Human β-Papillomavirus Types as Potentially Sex-Related and Modifiable Cancer Risk Indicators
Source: Cancers (Basel). 2025 Sep 16;17(18):3024. doi: 10.3390/cancers17183024 (PMC12468992; doi:10.3390/cancers17183024)
Supplement: Supplementary file 1 [file cancers-17-03024-s001.zip › Supplemetal Figure S2 .pdf]

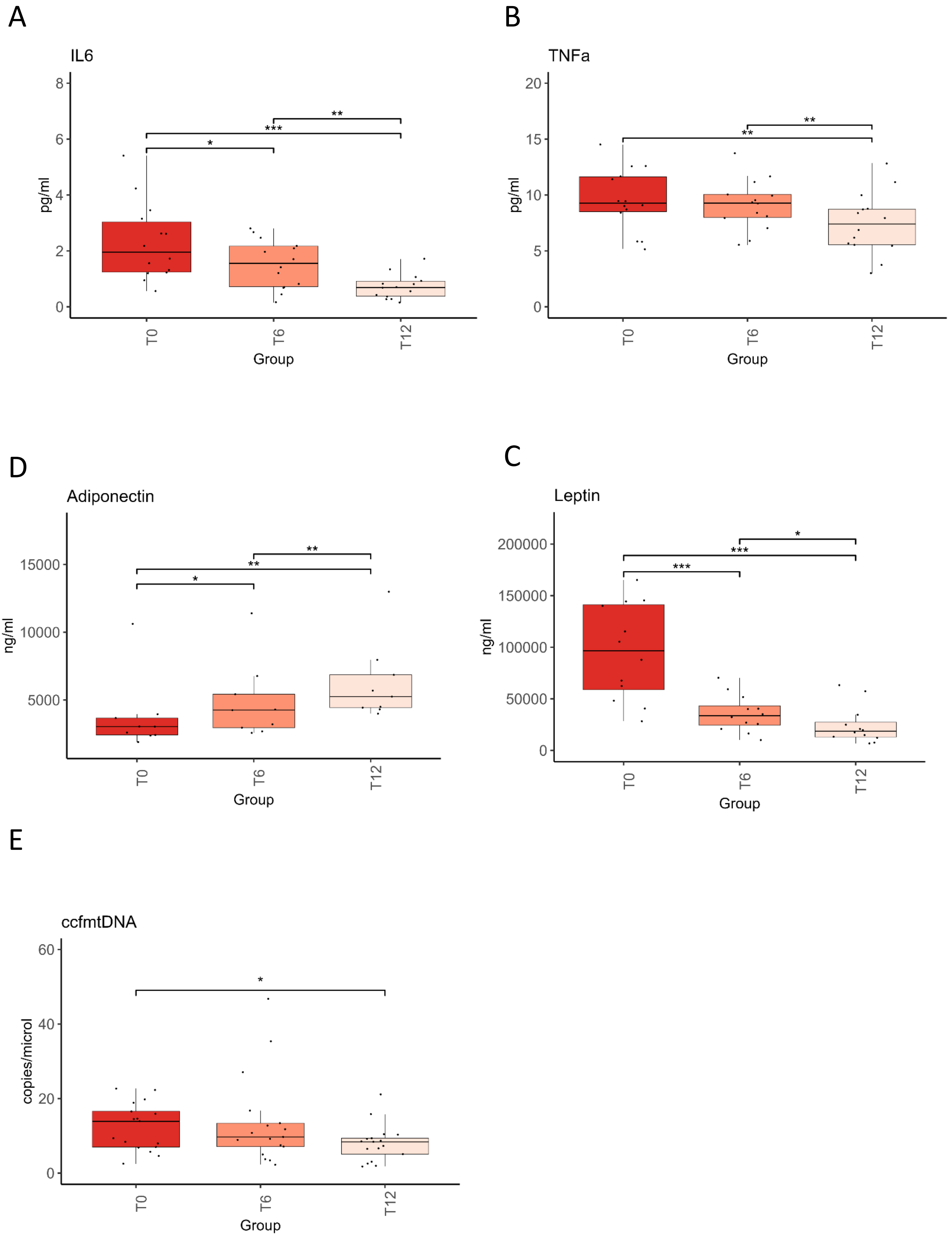

A
IL6
pg/ml
8
6
4
2
0
*
***
**
T0
T6
T12
Group
B
TNFa
pg/ml
20
15
10
5
0
**
**
T0
T6
T12
Group
D
Adiponectin
ng/ml
15000
10000
5000
*
**
**
T0
T6
T12
Group
C
Leptin
ng/ml
200000
150000
100000
50000
0
***
***
*
T0
T6
T12
Group
E
ccfmtDNA
copies/microl
60
40
20
0
*
T0
T6
T12
Group

Supplemetal Figure S2: Analysis of inflammation markers in individuals with obesity, before and after bariatric surgery.

All subjects were positive for β-HPV infection before surgery (T0). Plasma levels of (A) IL-6 (N=14), (B) TNF-α (N=17), (C) leptin (N=12), (D) adiponectin (N=9), and (E) circulating cell-free mtDNA (ccf-mtDNA) (N=17) are reported before (T0) and six (T6), and 12 months (T12) after bariatric surgery. A paired t-test was performed if the requirements of normal distribution (Shapiro-Wilk test) were met. Otherwise, the Wilcoxon signed-rank test was performed. * $p < 0.05$; ** $p < 0.01$; *** $p < 0.001$; and **** $p < 0.0001$.

Data redrawn from doi: 10.1038/s41419-024-06922-0.
